# AMP-18 Targets p21 to Maintain Epithelial Homeostasis

**DOI:** 10.1371/journal.pone.0125490

**Published:** 2015-04-28

**Authors:** Peili Chen, Yan Chun Li, F. Gary Toback

**Affiliations:** Department of Medicine, University of Chicago, Illinois 60637, United States of America; Indiana University School of Medicine, UNITED STATES

## Abstract

Dysregulated homeostasis of epithelial cells resulting in disruption of mucosal barrier function is an important pathogenic mechanism in inflammatory bowel diseases (IBD). We have characterized a novel gastric protein, Antrum Mucosal Protein (AMP)-18, that has pleiotropic properties; it is mitogenic, anti-apoptotic and can stimulate formation of tight junctions. A 21-mer synthetic peptide derived from AMP-18 exhibits the same biological functions as the full-length protein and is an effective therapeutic agent in mouse models of IBD. In this study we set out to characterize therapeutic mechanisms and identify molecular targets by which AMP-18 maintains and restores disrupted epithelial homeostasis in cultured intestinal epithelial cells and a mouse model of IBD. Tumor necrosis factor (TNF)-α, a pro-inflammatory cytokine known to mediate gastrointestinal (GI) mucosal injury in IBD, was used to induce intestinal epithelial cell injury, and study the effects of AMP-18 on apoptosis and the cell cycle. An apoptosis array used to search for targets of AMP-18 in cells exposed to TNF-α identified the cyclin-dependent kinase inhibitor p21^WAF1/CIP1^. Treatment with AMP-18 blunted increases in p21 expression and apoptosis, while reversing disturbed cell cycle kinetics induced by TNF-α. AMP-18 appears to act through PI3K/AKT pathways to increase p21 phosphorylation, thereby reducing its nuclear accumulation to overcome the antiproliferative effects of TNF-α. In vitamin D receptor-deficient mice with TNBS-induced IBD, the observed increase in p21 expression in colonic epithelial cells was suppressed by treatment with AMP peptide. The results indicate that AMP-18 can maintain and/or restore the homeostatic balance between proliferation and apoptosis in intestinal epithelial cells to protect and repair mucosal barrier homeostasis and function, suggesting a therapeutic role in IBD.

## Introduction

An agent that maintains and/or restores the homeostatic balance between proliferation and apoptosis in epithelial cells is essential to regulate gastrointestinal (GI) epithelial morphology and function to protect the mucosal barrier, and speed its recovery after injury. We have characterized a novel 18 kD protein isolated from the stomach, *A*ntrum *M*ucosal *P*rotein (AMP)-18, also known as gastrokine-1, whose pleiotropic properties suggest it could be developed into a new therapeutic agent to protect and heal the injured GI mucosa in patients with inflammatory bowel diseases (IBD) [1–4]. AMP-18 is synthesized in gastric antrum mucosal epithelial cells, stored in cytoplasmic granules, and secreted with mucus onto the apical cell surface. It appears to mediate its effects on intestinal and colonic epithelial cells by binding to the cholecystokinin-B/gastrin receptor (CCKBR) and activating its downstream pathways including Rho, ERK and p38 mitogen-activated protein kinases (MAPKs) [1,4,5]. Recombinant human (rh) AMP-18, and a synthetic AMP peptide comprised of amino acids 77–97 of the mature protein, each stimulate growth of epithelial cells, but not fibroblasts, and increase restitution of scrape-wounded gastric epithelial monolayers, suggesting important roles for AMP-18 in maintaining integrity of the mucosal barrier through restitution and cell growth following injury [3,5]. AMP-18 also facilitates translocation and assembly of multiple proteins into tight junctions (TJs) and their association with and subsequent stabilization of the actin filament network [1]. The TJ consists of multiple proteins that bind epithelial cells together at their apical surface, and is essential to create and maintain mucosal barrier function. In addition, an anti-apoptotic effect of AMP-18 has been observed in epithelial cells exposed to tumor necrosis factor (TNF)-α, which could be another protective mechanism that facilitates recovery of disrupted barrier structure and function [6]. In the dextran sulfate sodium (DSS)-induced mouse model of colonic injury, treatment with AMP peptide protected against bloody diarrhea and loss of weight, preserved colon length, and reduced the development of mucosal erosions [1]. Thus, AMP-18 appears to be a promising new agent that could heal the injured intestinal epithelium.

Ulcerative colitis (UC) and Crohn’s Disease (CD) are chronic inflammatory disorders that occur in genetically predisposed patients with abnormal intestinal epithelial function and homeostasis in response to bacterial pathogens [7,8]. Increasing evidence suggests that intestinal epithelial cells (IEC) have multiple functions including creating and maintaining a physical barrier and controlling its permeability, producing mucus and regulating its composition, and serving as non-professional antigen presenting cells by processing and presenting antigens directly to cells of the intestinal immune system [9]. To perform these diverse functions, epithelial cells communicate with their surrounding microenvironments and cells through converging integrated signaling cascades to maintain integrity of the barrier, which is essential for preventing pathological entry of food-derived antigens, microorganisms and their toxins into GI tissues [7]. When the epithelial barrier is disrupted, the resulting increase in mucosal permeability can allow toxins to pass from the gut lumen into the submucosa, triggering an aberrant immune response that can result in development of IBD.

Several changes in barrier function have been identified in IBD which include impaired epithelial junctions, altered mucus components [8] and expression of toll-like receptors (TLR) [10,11], NOD2 gene mutations, and epithelial cell dysfunction [12]. These findings point to the importance of epithelial cells as a prominent therapeutic target in patients with IBD. Rather than being a static, impregnable layer, the epithelial barrier is a highly dynamic structure that undergoes consistent rapid renewal by adjusting cell proliferation, differentiation and death in response to a variety of intrinsic and extrinsic signals. A well-regulated balance between proliferation and apoptosis in mucosal epithelial cells is essential to maintain normal homeostasis and barrier function [13,14], whereas chronically dysregulated homeostasis is speculated to be responsible for barrier dysfunction in IBD [15–18]. Increased apoptosis and dysregulated proliferation of the colonic epithelium have been reported in animal models of IBD [16,19–21].

Inflammation has devastating effects on intestinal epithelial function and homeostasis in which pro-inflammatory cytokines play an important role. TNF-α is a key regulator of immunological responses, and its aberrant production underlies the pathogenesis of many human diseases, in particular acute and chronic inflammatory diseases [22], for which TNF-blocking agents have become an established treatment, as for IBD [23]. Barrier dysfunction in IBD is closely associated over-production of inflammatory cytokines such as TNF-α, interferon (IFN)-γ, and interleukin (IL)-13 due to uncontrolled immune responses. TNF- ↦ has been shown in many studies to disrupt epithelial barrier structure and increase mucosal permeability, at least in part by disrupting TJ structure and function between epithelial cells [24]. In cultures of the colonic epithelial cell line Caco-2, TNF-α increases monolayer permeability and flux of small molecules within 24 h of treatment, and reduces transepithelial electrical resistance (TER) after 48 h. This increased permeability is accompanied by a decrease in the TJ protein, ZO-1. Exposure to this cytokine was also associated with internalization of TJ transmembrane proteins, such as JAM-1, occludin, and claudin-1/4; the detergent solubility profiles of JAM-1 and E-cadherin, and their affiliation with "raft-like" membrane microdomains, were modified as well. *In vivo* administration of TNF-α results in occludin endocytosis and increased epithelial permeability [25]. In addition, TNF-α can induce apoptosis in the epithelium which may contribute to disruption of mucosal integrity and barrier function. In patients with IBD, increased apoptosis has been found in the acute inflammatory sites throughout the entire crypt-villus axis in contrast to apoptosis normally restricted to the apical aspect of the villus. Apoptosis/proliferative rates were found to increase significantly in line with the inflammatory process [26]. Increased IEC apoptosis in chronic UC is associated with elevated TNF-a. The introduction of anti-TNF agents was a breakthrough in the management of IBD, as these biologics can inhibit IEC apoptosis [27,28], rapidly induce mucosal healing and restore intestinal mucosal barrier function, thereby inducing remission.

The aim of this study is to characterize therapeutic mechanisms by which AMP-18 can restore and maintain homeostasis in cultured intestinal epithelial cells and an animal model of IBD; specifically to identify molecular targets of AMP-18 that mediate its cell proliferative and anti-apoptotic effects. IECs undergo vigorous turnover through consistent and balanced proliferation and apoptosis along the crypt-villus axis [29]. Thus, the balance between apoptosis and proliferation must be strictly maintained to sustain tissue homeostasis. In somatic cells, apoptosis and cell proliferation are linked by cell-cycle regulators and apoptotic stimuli that affect both processes. Cell cycle progression is controlled by complexes formed by specific cyclins and cyclin-dependent kinases (CDKs) through different phases of the cell cycle, and are negatively regulated by CDK inhibitors such as p21^WAF1/CIP1^ (subsequently referred to as p21) [30]. p21 is one of the best described members of the Cip/Kip family of CDK inhibitors. It binds to and inhibits the activity of multiple cyclin/CDK complexes throughout the cell cycle. In addition, p21 also plays an important role in apoptosis, terminal differentiation, and cellular senescence [31–34]. In the present study we found that by targeting p21, AMP-18 appears to maintain tissue homeostasis during protection and repair of injured intestinal epithelial cells.

## Materials and Methods

### Materials

Chemically synthesized AMP peptide (LDALVKEKKLQGKGPGGPPPK), a scrambled peptide (GKPLGQPGKVPKLDGKEPLAK), and recombinant human (rh)AMP-18 were prepared by GenScript (Piscataway, NJ) as described previously [5]. RhAMP-18 was expressed and purified as a His_6_-tagged fusion protein. Briefly, the coding sequence for full-length human AMP-18 was cloned into an *E*. *coli* expression vector, pGSE3, and the expressed protein was purified from 5 L of culture medium by affinity column chromatography. AMP peptide and rhAMP-18 were found to be equally effective (data not shown), as previously reported (1, 15, 16) and therefore both were used. Cell culture medium, fetal bovine serum (FBS), and penicillin and streptomycin were obtained from Gibco BRL, Life Technologies (Gaithersburg, MD). Total p21, phosphorylated p21 (ser 146), and Alexa Fluor 647 conjugated-p21 antibodies were obtained from Santa Cruz Biotechnology (Dallas, TX); TNF-α from PeproTech (Rocky Hill, NJ); and other reagents from Sigma-Aldrich unless otherwise specified.

### Cell Cultures

Nontransformed IEC-18 epithelial cells (ATCC) derived from normal rat ileum were used to model the GI epithelium. Cells were grown in Dulbecco’s modified Eagle medium (DMEM) with 10% (vol/vol) FBS, penicillin (50 U/mL) and streptomycin (50 μg/mL), (Gibco BRL) at 37°C in a humidified incubator supplemented with 5% CO_2_. When treated with TNF-α in the presence or absence of rhAMP-18 or AMP peptide, cells were serum starved in DMEM with 0.5% FBS for at least 3 to 6 h.

### Sodium Dodecyl Sulfate-Polyacrylamide Gel Electrophoresis (SDS-PAGE) and Immunoblotting

To prepare cell lysates, cultures were rinsed and then harvested in iced phosphate buffered saline (PBS) by scraping the monolayer with a cell lifter. The detached cells were pelleted at 4°C and extracted on ice for 30 min in lysis buffer (50 mM Tris-HCl, pH 7.4, 1% Nonidet P-40, 0.25% sodium deoxycholate, 150 mM NaCl, 100 mM NaF, 10% glycerol, 10 mM EDTA) containing protease and phosphatase inhibitors (2 mM sodium orthovanadate, 1 mM phenylmethylsulfonyl fluoride, 50 μg/ml antipain, 1 μg/ml aprotinin, 1 μg/ml leupeptin, and 1 μg/ml pepstatin). Cell lysates were clarified by centrifugation at 14000 × *g* for 15 min at 4°C. Protein concentration was determined by BCA assay (Pierce). For immunoblotting assays, 30 to 50 μg protein/lane was resolved by SDS-PAGE, transferred onto Immobilon membranes (Millipore, Bedford, MA) followed by blocking with 5% bovine serum albumin in TBST buffer (20 mM Tris-HCl, 150 mM NaCl, 0.1% Tween 20 at pH 7.5), and incubated with designated antibodies. After incubation with horseradish peroxidase (HRP)-conjugated secondary antibodies, immunoreactive bands were visualized using chemiluminescence (ECL, Amersham Biosciences). When reprobed, blots were first stripped with a buffer containing 50 mM Tris-HCl, pH 6.8, 2% SDS, and 0.1 M 2-mercaptoethanol. Images were analyzed by densitometry. The immunoblot shown in each figure represents one of at least three experiments.

### Cell Cycle and p21 Expression Analyzed by Flow Cytometry

Cell monolayers were rinsed with PBS, detached with Accutase cell detachment solution (BD Biosciences), and washed twice with cold PBS before fixation with 3% paraformaldehyde on ice for 30 min. After washing with PBS twice, cells were permeabilized with cold methanol. DNA content was analyzed by staining with 40 μg/ml propidium iodide (PI) in the presence of 100 μg/ml DNAse-free RNAse A. The level of p21 protein was assessed with Alexa Fluor 647 conjugated-p21 antibody. Stained cells were analyzed by FACScanto (BD Biosciences) using FACSDiva (BD Biosciences).

### Chemically-Induced Colitis Model and AMP Peptide Treatment

Mice were housed in a barrier facility, exposed to a 12-h light/dark cycle, and food and water were provided *ad libitum*. Colitis was induced using 2,4,6-trinitrobenzenesulfonic acid (TNBS), as described previously, in 8 to 12 week-old wild type (wt) (n = 15) and vitamin D receptor knockout (VDR^-/-^) mice (n = 15) [35]. VDR^-/-^ mice are particularly susceptible to DSS- or TNBS-mediated injury and develop very severe colitis leading to high mortality with high reproducibility and predictability [35,36]. Overnight-fasted mice were treated, under anesthesia, with 100 mg/kg TNBS (Sigma-Aldrich) dissolved in 50% ethanol via intra-rectal injection using a 1-ml syringe fitted with an 18-gauge stainless steel gavage needle; 50% ethanol treatment served as a control. Body weight, stool consistency, and GI bleeding were monitored daily throughout the study. AMP peptide (25 mg/kg body weight) or the vehicle (PBS) was administered intraperitoneally once daily for 5 days before animals were given TBNS, and continued thereafter for 5 days. Clinical scores and colonic damage scores were estimated as detailed previously [35,37,38]. Animals that lost greater than 20% of their starting body weight would have been euthanized with CO_2_, but none did. Five days after TNBS administration, animals were sacrificed by cervical dislocation after euthanasia with CO_2_, and colons were collected and fixed immediately. Colonic histological analyses were performed using the “Swiss roll” method or “bread-loafing” cross sections. These studies were approved by the University of Chicago Animal Care and Use Committee.

### Histology and Immunohistochemical Staining

Freshly dissected colons were fixed overnight with 4% formaldehyde in PBS (pH 7.2), processed, and embedded in paraffin wax. Tissues were cut into 4-μm sections, and stained with hematoxylin and eosin, or anti-p21 antibodies (Santa Cruz Biotechnology, Inc.) followed by staining with HRP-conjugated anti-IgG as second antibodies. Antigens were then visualized with 3, 3′-diaminobenzidine substrate (Sigma-Aldrich) and observed under a light microscope.

## Results

### p21 Induction by TNF-α is Inhibited by AMP-18

We have reported that AMP-18 inhibits apoptosis induced by TNF-α in epithelial cells, and that AMP peptide is protective and can speed healing in mice when colonic injury is induced by DSS [1,4,6]. To identify molecular target(s) of AMP-18 we used an apoptosis array (R & D Systems) to compare apoptosis-related proteins in IEC-18 cells exposed to TNF-α in the presence or absence of AMP-18 (data not shown). This array assay allowed us to detect the relative levels of 35 apoptosis-related proteins simultaneously that may be affected by AMP-18 treatment. Of these 35 proteins, p21 and cleaved caspase 3 (as a marker for apoptosis) were elevated in cells exposed to TNF-α alone, but significantly reduced when cells exposed to the cytokine were treated with AMP-18.

**Fig 1:**
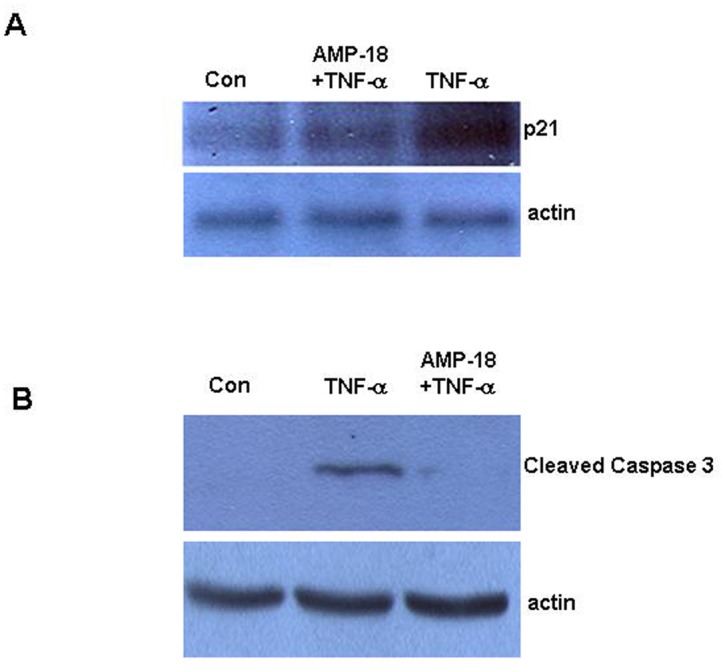
Pretreatment with AMP-18 blocks TNF-↦ induction of p21 and cleavage of caspase 3. IEC-18 cells were untreated (Con), or treated with rhAMP-18 (2 ∓g/mL) for 30 min before exposure to TNF- ↦(50 ng/mL) for 2 h. Expression level of total p21 (*Panel* **A**) and cleaved caspase 3 (*Panel* **B**.) were detected by immunoblotting. Actin was assessed as a loading control. Representative results of 3 independent experiments are shown.

p21 is one of the best described members of the Cip/Kip family of cyclin-dependent kinase (CDK) inhibitors whose function has been implicated in cell cycle regulation, differentiation, apoptosis and senescence [33]. As shown by immunoblotting in Fig 1A, the level of p21 protein was increased 4.9-fold in IEC-18 cells exposed to TNF-α. Pretreatment with AMP-18 significantly inhibited this induction by 44% (P = 0.02). The down-regulation of p21 by AMP-18 could contribute to its growth-promoting activity on epithelial cells we reported previously [2,3,5]. Induction of p21 by TNF-α also suggests a mechanism by which TNF-α elicits its pro-apoptotic effect on permissive cells. Indeed, as suggested in the apoptosis array assay, we found that cleaved caspase 3 was induced by 7.7-fold in cells exposed to TNF-↦ whereas treatment with AMP-18 inhibited TNF-↦induced cleavage of caspase 3 by 73% (Fig 1B). No significant change in p21 or cleaved caspase 3 was observed in cells treated with AMP-18 alone (data not shown). Therefore, TNF-α can increase the level of p21 protein and could induce apoptosis in intestinal epithelial cells, whereas pretreatment of cells with AMP-18 can suppress these effects of TNF-α, suggesting that p21 could be a critical target by which AMP-18 limits apoptosis and thereby restores homeostasis in the epithelial barrier.

### AMP-18 Reverses Apoptotic and Antiproliferative Effects of TNF-α

TNF-α is a pleiotropic cytokine that can exert antiproliferative and proapoptotic effects in different types of cells [39,40], and is an important mediator of GI mucosal injury in IBD. It has been shown to induce apoptosis in IEC-18 cells [41].

**Fig 2:**
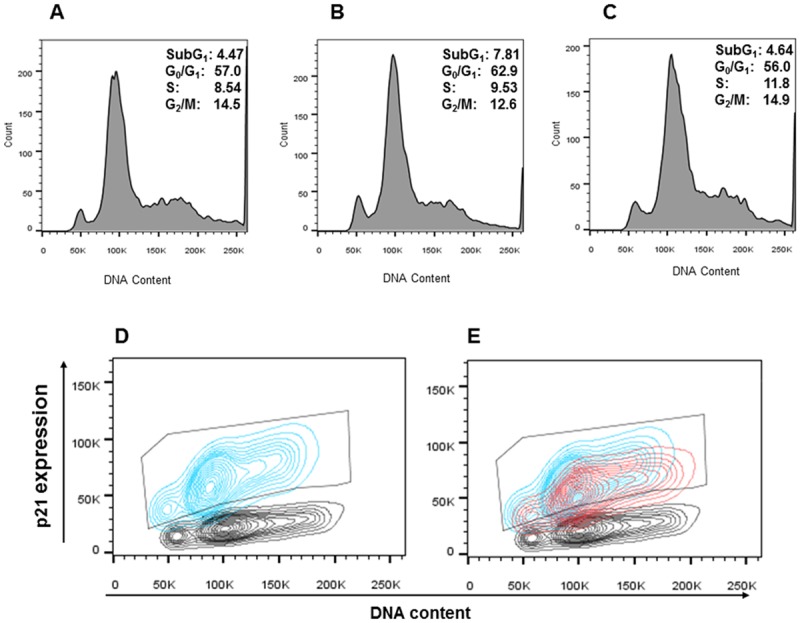
Effects of AMP-18 treatment on cell cycle. IEC-18 cells were untreated (*Panel* **A**), treated with TNF-↦ (50 ng/mL) alone for 2 hr (*Panel* **B**), or with rhAMP-18 (2 ∓g/mL) for 30 min before exposure to TNF- ↦(50 ng/mL) (*Panel* **C**). Then cells were harvested, fixed and permeabilized before staining for DNA content with PI for cell cycle analysis. Double staining with PI showing DNA content at different phases of cell cycle, and Alexa Fluor 647 conjugated antibody to measure p21 expression level are depicted in panels **D** and **E**. *Gray* contours: untreated cells; *blue* contours: cells exposed to TNF-α alone; *red* contours: cells treated with rhAMP-18 before exposure to TNF-α. The gate in panels **D** and **E** contains cell populations with increased p21 expression. Representative results of 3 independent experiments are shown.

To characterize the effects of AMP-18 on the antiproliferative and proapoptotic effects induced by TNF-α, the cell cycle was analyzed in IEC-18 cells in the presence of AMP-18 and/or TNF-α using flow cytometry. IEC-18 cells were exposed to 50 ng/ml TNF-α with or without AMP-18 for 6 h and different phases of cell cycle were identified by PI staining of cellular DNA. In control cells that were serum starved, a small subG1 fraction (4.47%) was observed, representing a low level of apoptosis (Fig 2A). The majority of cells were in G0/G1 phase (57.0%). TNF-α induced a >60% increase in subG1 cells (7.81%, P<0.001), and >10% increase in G1 phase cells (62.9%, P<0.01), indicating proapoptotic and antiproliferative effects (Fig 2B). In contrast, treatment with AMP-18 completely reversed cell cycle changes induced by TNF-α, seen in subG1 and G0/G1 cells (4.64% and 56.0%, respectively) back to the control level (Fig 2C).

The relationship between p21 expression and the cell cycle was investigated by double staining cells with Alexa 647 conjugated antibody to total p21, and PI to depict p21 protein levels during different phases of the cell cycle (Fig 2D and 2E). p21 expression (Fig 2D, *blue* contours) increased in 93.5% of cells displayed as the population within the gate at all phases of the cycle following exposure to TNF-α (including the subG1 phase) compared to untreated cells (*gray* contours), suggesting that p21 might mediate the proapoptotic and antiproliferative effects induced by TNF-α.

Treatment with AMP-18 in the presence of TNF-α decreased p21 expression in all phases of cell cycle (Fig 2E, *red* contours) compared to TNF-α alone (*blue* contours) by 16% (P<0.04) of the gated population. This suggested that p21 could serve as a therapeutic target by which AMP-18 antagonizes TNF-α induced proapoptotic and antiproliferative effects. Indeed, our previous studies [6], and Fig 1B demonstrate that AMP-18 is antiapoptotic.

### Pathways Induced by AMP-18 that Regulate p21 Function

**Fig 3:**
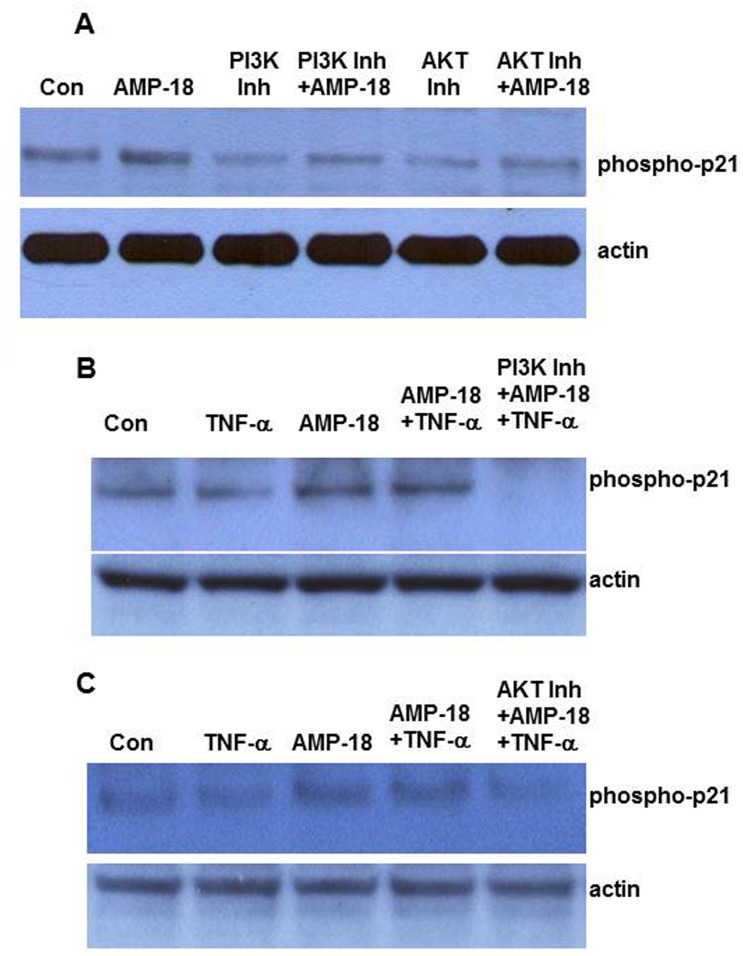
PI3K/AKT pathways mediate AMP-18-induced phosphorylation of p21. Cells were untreated (Con); treated with rhAMP-18 (2 μg/mL), PI3K inhibitor (Inh) (LY294002, 10 μM), AKT inhibitor (VIII, 2 μM) alone, or a combination of AMP-18 with either of the inhibitors (*Panel* **A**); or treated with TNF-α (50 ng/mL), AMP-18 alone, or TNF-α and AMP-18 in the presence or absence of PI3K inhibitor (*Panel* **B**); or AKT inhibitor (*Panel* **C**). When cells were exposed to multiple agents, they were pre-treated with either inhibitor for 30 min, followed by treatment with rhAMP-18 for 30 min before final exposure to TNF-α for 2 h. Phosphorylation of p21 was determined by immunoblotting with a phospho-p21 antibody, and equal loading was verified by immunoblotting of actin. Representative results from 3 independent experiments are shown.

Function and expression of p21 are regulated at transcriptional and post-translational levels through mechanisms including protein phosphorylation, stabilization, and ubiquitination [30,31]. We set out to determine which pathway(s) mediates AMP-18 regulation of p21. Our previous observations indicate that AMP-18 activates several signaling pathways such as MAPK (ERK, JNK and p38), PI3K/AKT, and RhoA [5]. p21 is known to be regulated by several pathways including MAPK and PI3K/AKT [42]. To investigate which of these pathways mediates regulation of p21 by AMP-18, cells were treated with TNF-α or AMP-18 alone, or together in the presence of different inhibitors that target the specific pathways (U0126 for ERK, SB203580 for p38, JNK inhibitor for JNK, LY294002 for PI3K, and AKT Inhibitor VIII for AKT). We found that the PI3K inhibitor or AKT inhibitor was able to significantly inhibit p21 phosphorylation (Fig 3A), while treatment with MAPK inhibitors showed negligible effects on p21 phosphorylation (data not shown). These findings are consistent with the notion that the PI3K/AKT pathway targets p21 to confer antiapoptotic and prosurvival signals, resulting in increased S phase entry, growth promotion, and resistance to death signals [42]. AKT-mediated phosphorylation of p21 is associated with its cytoplasmic localization and increased stability [42,43]. We therefore explored the effects of these two inhibitors on p21 phosphorylation in the presence of AMP-18 or TNF-α alone, or in combination. As shown in Fig 3A, treatment with AMP-18 induced a 26% increase in p21 phosphorylation (P = 0.002). When PI3K inhibitor or AKT inhibitor was present, AMP-18 induced phosphorylation was reduced by 48% and 35%, respectively. That both PI3K and AKT inhibitors reduce AMP-18 mediated phosphorylation of p21 confirms our previous observation that AMP-18 stimulates PI3K/AKT (data not shown). Exposure to TNF-α alone, slightly but consistently reduces phosphorylation of p21 by 24% (P = 0.012, Fig 3B and 3C).

When cells were exposed to either the PI3K or AKT inhibitor followed by AMP-18 and TNF-α, phosphorylation of p21 was reduced further by 81% (P = 0.003) and 56% (P = 0.02), respectively (Fig 3B and 3C). These results suggest that AMP-18 may, at least in part, target PI3K/AKT pathways to regulate p21 phosphorylation, although other pathways could also be involved.

### AMP-18 Induced Subcellular Redistribution of p21 Protein

**Fig 4:**
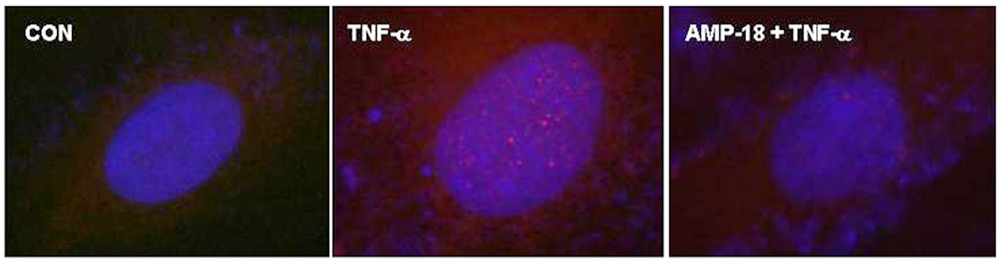
Pretreatment with AMP-18 reduces TNF-↦ induced nuclear translocation and accumulation of p21. Cells were untreated (CON), treated with TNF-↦ (50 ng/mL) alone, or with rhAMP-18 (2 ∓g/mL) for 30 min before exposure to TNF-↦ (50 ng/mL) (AMP-18 + TNF-↦*)* for 2 h. Subcellular localization of p21 (*red*) was visualized by immunofluorescence. The nucleus was counterstained with DAPI (*blue*). TNF-↦ induced nuclear accumulation of p21 (*center panel*) was reduced by pretreatment with AMP-18 (*right panel*). Representative results from 3 independent experiments are shown.

Subcellular localization is another important mechanism that regulates function of p21 [44]. p21 and other Cip/Kip family CDK inhibitors share a C-terminal nuclear localization signal that appears to enable it to translocate between the cytoplasm and nucleus. Different functions are exhibited by p21 in cytoplasm and nucleus, likely due to specific targets in each location. It has been reported that cytoplasmic localization of p21 promotes cell growth and protects cells against apoptosis, whereas nuclear localization is associated with inhibition of cell cycle progression [45,46]. Therefore, we sought to determine if AMP-18 affects subcellular localization of p21 protein. As shown in Fig 4, *left panel*, control cells demonstrated only scattered occasional staining of p21 (*red*) in the nucleus (*blue*). TNF-α markedly increased accumulation of p21 protein within the nucleus which appears in a granular pattern (*middle panel)*. Pretreatment with AMP-18 substantially reduced nuclear accumulation of p21, so that only scattered staining was visible in nucleus and cytoplasm (*right panel*). These observations suggest that AMP-18 can reduce TNF-α mediated nuclear accumulation of p21, thereby stimulating cell growth.

### Role of p21 in AMP Peptide Treatment of TNBS Colitis

**Fig 5:**
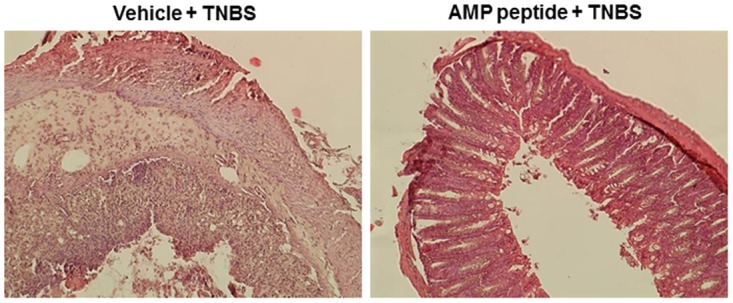
Therapeutic effect of AMP peptide in mice with TNBS colitis. Colitis was induced using TNBS in VDR^-/-^ mice (n = 15). Treatment with vehicle (PBS) (*left panel*) or AMP peptide (25 mg/kg, i.p.) (*right panel*) was initiated once daily 5 days before exposure to TNBS and continued 5 days afterwards. Colons were collected, fixed and subjected to histological analyses as described in Materials and Methods. Representative images from a total of 15 animals are shown.

To investigate the role of p21 in maintaining mucosal epithelial homeostasis in AMP-mediated protection of epithelial cells *in vivo*, we studied expression of p21 in TNBS-induced colitis in vitamin D receptor (VDR)-deficient (VDR^-/-^) mice. Emerging evidence suggests that the VDR plays a critical role in mucosal barrier homeostasis by preserving the integrity of cell junction complexes and the healing capacity of colonic epithelium [35,36,47]. Genetic VDR deletion in mice is associated with normal histology but leads to increased vulnerability to TNBS- or DSS-induced colitis [35,36]. VDR^-/-^ mice given TNBS exhibit a greater loss of intestinal transepithelial electric resistance (TER), disruption of epithelial junctions, and typical IBD symptoms such as severe diarrhea, rectal bleeding and marked body weight loss, as well as increased mortality. Histological examination revealed extensive ulceration and impaired wound healing in the colonic epithelium in the VDR^-/-^ mice [35], along with crypt hyperplasia, loss of crypts, severe focal ulceration, thickened colonic walls and severe inflammation (Fig 5). In addition, VDR knockdown in cultured Caco-2 cell monolayers with small interfering (si) RNA reduced junction proteins and TER [36]. Therefore, vitamin D deficiency compromises the mucosal barrier leading to increased susceptibility to mucosal damage and increased risk of IBD. To evaluate the therapeutic efficacy of AMP peptide in TNBS colitis, wt and VDR^-/-^ mice were given the peptide (25 mg/kg, i.p.) once daily for 5 days before receiving TNBS. Treatment with AMP peptide was continued for 5 days after TNBS, while control animals received the vehicle (PBS). AMP peptide treatment did not demonstrate a significant survival benefit in 5 wt mice, which was 80%, as observed in previous studies [35]. In contrast, all 5 VDR^-/-^ mice treated with vehicle died by day 5 after TNBS, as reported previously [35], whereas treatment of 4 VDR^-/-^ mice with AMP peptide resulted in 50% survival. The capacity of AMP peptide to preserve colonic mucosal integrity and prevent transmural inflammation in these animals is depicted in Fig 5.

**Fig 6:**
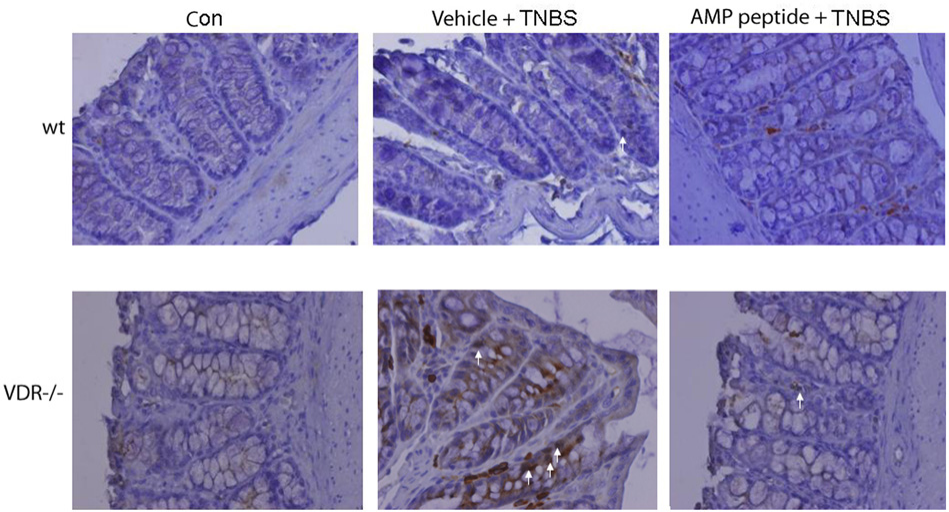
Role of p21 in AMP peptide treatment of TNBS colitis. TNBS was used to induce colitis in wild type (*3 top panels*) and VDR^-/-^ mice (*3 bottom panels*). Untreated wild type and VDR^-/-^ animals served as controls (con) (*left panels*). Wild type and VDR^-/-^ mice given TNBS were treated with vehicle (PBS) (*center panels*). In mice receiving AMP peptide (25 mg/kg, i.p.) treatment was initiated once daily 5 days before exposure to TNBS and continued 5 days afterwards (day 10) (*right panels*). Total p21 (white arrows) was analyzed by immunohistochemistry with anti-p21 antibody in freshly dissected and 4% formaldehyde-fixed colon tissues from wild type (*top panels*) and VDR^-/-^ mice (*bottom panels*). Representative images from a total of 30 animals are shown.

p21 protein levels were assessed by immunohistochemistry in freshly-dissected and 4% formaldehyde-fixed colon tissues. Expression of p21 protein in untreated wt control (con) and VDR^-/-^ mouse colon was not readily detectable (Fig 6, *left panels*). Staining of p21 was occasionally observed in wt mice given TNBS (*top*, *middle panel*), whereas treatment with AMP peptide appeared to reduce the number of p21 positive cells, although it was not possible to measure this accurately because of the small number of p21 positive cells (*top*, *right panel*). In contrast, p21 positive cells became numerous in VDR^-/-^ mice given TNBS, whereas treatment with AMP peptide remarkably reduced the number of p21-stained cells (*bottom middle and right panels*, respectively). These data indicate that AMP peptide can reverse induction of p21 *in vivo* (by TNBS) as well as in cell culture (by TNF-α), thereby alleviating growth inhibition and allowing progression of the cell cycle to repair the injured mucosa.

## Discussion

In this study we set out to identify new molecular targets and mechanisms that mediate the therapeutic efficacy of AMP-18. Treatment with AMP-18 reduced p21 expression (Fig 1A), apoptosis (Fig 1B) [6], and disturbed cell cycle kinetics in epithelial cells induced by the cytokine TNF-α, a known mediator of GI mucosal barrier injury (Fig 2). AMP-18 appears to act through PI3K/AKT pathways (Fig 3) to reduce nuclear accumulation (Fig 4) of p21, thereby overcoming the antiproliferative effects of TNF-α. In VDR^-/-^ mice with TNBS-induced IBD, the increase in p21 expression in colonic epithelial cells was suppressed by treatment with AMP peptide (Fig 6). By targeting p21, AMP peptide could maintain and/or restore the homeostatic balance between proliferation and apoptosis in intestinal epithelial cells to protect and repair disrupted mucosal barrier homeostasis and function.

p21 is the best described member of the Cip/Kip family of cyclin-dependent kinase (CDK) inhibitors. When localized in the nucleus, p21 forms a quaternary complex with cyclins (A, B, D or E), CDKs (1, 2 or 4), and proliferating cell nuclear antigen (PCNA), and functions as a key regulator of progression at the G1/S phase of the cell cycle in response to various exogenous and endogenous factors. In addition to its primary role as an inhibitor of the cell cycle, p21 is also implicated in terminal differentiation, cellular senescence, and apoptosis [31–34], although its role in apoptosis is highly dependent on cellular context [32,48–51].

Function and expression of p21 can be regulated by multiple pathways at transcriptional and post translational levels through mechanisms involving phosphorylation, subcellular localization, RNA stabilization, and ubiquitination [31,43,44,52]. p21 expression can be induced through p53-dependent or-independent pathways by growth factors or cytokines such as TNF-α. When TNF-α has been shown to induce cell cycle arrest and block proliferation in specific cells, the G1/S checkpoint appears to be an important target of its effect on cell cycle regulation [53]. When localized in the cytoplasm, p21 protects cells against apoptosis by forming a complex with Rho kinase 2 (Rock2) and apoptosis signal regulating kinase 1 (ASK1), and acts as an inhibitor of stress fiber formation [45,46]. In addition, this cytoplasmic localization is closely correlated with phosphorylation of p21 that appears to be mediated by the AKT pathway [42,43]. In the present study, AMP-18 reduced nuclear accumulation of p21 which suggests at least one mechanism by which AMP-18 could exert its anti-apoptotic and growth-promoting effects in the presence of TNF-α.

Dysregulated apoptosis/proliferation in epithelial cells has been observed in patients and animal models of IBD [26]. This disturbed homeostasis in IBD is considered, at least in part, to be a result of increased cytokine production (e.g., TNF-α) by over-activated immune cells, which can induce apoptosis in the epithelium and thereby contribute to disruption of mucosal integrity and barrier function. Current therapy targets the increased cytokine production and inflammation utilizing a TNF-neutralizing antibody (infliximab) which benefits these patients. Anti-TNF antibody treatment effectively reduces epithelial cell apoptosis and increases mucosal repair, accompanied by reduced Fas/CD95 expression in the intestinal epithelium of mice [27]. Furthermore, in the acute DSS colitis mouse model, anti-TNF treatment reduced expression of gene targets that might mediate epithelial cell injury; and in chronic DSS colitis it abrogated elevated levels of cleaved caspases 3 and 9, highlighting the importance of apoptosis in these conditions [16].

The participation of cell apoptosis in the maintenance of intestinal epithelial barrier functions has been controversial in the literature [54,55]. Under normal conditions, balanced homeostasis between proliferation and apoptosis is maintained, which allows a “normal” number of apoptotic cells to be shed without disrupting barrier function [56]. However, in IBD, overproduction of inflammatory cytokines, especially TNF-α, may cause a marked increase in apoptosis that is beyond the cell proliferation capacity of the tissue, thereby compromising barrier function and structure. For example, it was reported that TNF-α-induced apoptosis in HT-29/B6 cells accounts for 56% of the TNF-α-mediated increase in epithelial monolayer permeability [55]. Extensive apoptosis, as occurs in UC, can impair epithelial integrity [57]. An intact intestinal epithelium can be maintained after apoptosis of single cells; however, substantial apoptosis can compromise barrier function and crypt-villus architecture. An agent that maintains and/or restores the homeostatic balance between proliferation and apoptosis in epithelial cells, such as AMP peptide, would be of great therapeutic value to protect the mucosal barrier, and speed its recovery after injury in patients with IBD. In addition to its effects on cell homeostasis, to determine if AMP peptide also acts through other mechanisms, such as inhibition of cytokine production and activation of inflammatory cells in IBD [58], will require further study.

Dysregulation of epithelial homeostasis is closely related to loss of cell cycle control. It has been reported that high p21 expression and p53 accumulation are characteristic of the active and remission phases of UC [59]. A study that analyzed expression of p21 and other cell cycle-related proteins found that p21 expression was higher in colonic epithelial cells in patients with IBD compared to healthy controls, and p21 expression levels were correlated with disease activity [60]. In the present study we found that treatment with AMP-18 inhibited p21 expression and nuclear localization induced by TNF-α, and completely reversed the cell cycle disturbance and cell apoptosis, suggesting that p21 can serve as a therapeutic target through which AMP-18 antagonizes TNF-α induced proapoptotic and antiproliferative effects. However, treatment with AMP peptide would be untenable if it stimulates growth of tumor cells or inhibits antineoplastic therapies, a concern that was proven to be untrue in a xenograft model [6]. AMP-18/peptide appears to act differently on p21 and cell homeostasis in non-transformed cells (Figs 1, 2D and 2E) compared to gastric cancer cells in which transfection of AMP-18 cDNA increased the level of p21, inhibited cell proliferation, induced apoptosis [61], and inactivated the PI3K/AKT pathway [62].

In summary, epithelial homeostasis plays an essential role in maintaining intestinal barrier function that is important for a normal host defense against pathogens in the gut lumen while allowing transport of nutrients. In IBD, dysregulated epithelial homeostasis can occur between apoptosis and proliferation leading to increased barrier permeability. AMP peptide, which acts by targeting p21, could restore mucosal epithelial homeostasis and thereby provide a new therapeutic agent to treat patients with this condition.
